# Tropical vulnerability and temperate resilience: molecular and physiological insights into cold stress adaptation in oil palm

**DOI:** 10.1080/21645698.2026.2739106

**Published:** 2026-09-29

**Authors:** Qiufei Wu, Muhammad Imran, Guanming Chen, Xianhai Zeng, Lixia Zhou

**Affiliations:** a State Key Laboratory of Tropical Crop Breeding, Coconut Research Institute, Chinese Academy of Tropical Agricultural Sciences, Sanya/Wenchang, Hainan, China; b School of Breeding and Multiplication (Sanya Institute of Breeding and Multiplication), School of Tropical Agriculture and Forestry, Hainan University, Sanya, China

**Keywords:** Cold stress, molecular breeding, multi-omics integration, oil palm, stress tolerance mechanism

## Abstract

Cold stress is a major abiotic constraint limiting the growth and productivity of oil palm (*Elaeis guineensis*). Its high sensitivity to chilling causes physiological impairment, metabolic disruption, and reduced oil yield and quality. This review summarizes physiological, biochemical, and molecular mechanisms of cold stress responses in oil palm, with comparative insights from rapeseed and soybean. Key pathways, including Ca^2+^ signaling, MAPK cascades, and the ICE-CBF/DREB network, coordinate membrane lipid remodeling, osmotic adjustment, antioxidant defense, and hormonal regulation. Key genes (*EgCBF, EgICE1, EgNCED, EgFAD2, EgSAD, EgP5CS*) and transcription factors (*EgWRKY, EgNAC, EgMYB*) contribute to stress adaptation. However, these responses may be insufficient under prolonged or recurrent chilling, potentially disrupting lipid remodeling, water relations, and plant performance. Advances in GWAS, QTL mapping, multi-omics, genomic selection, and genome editing provide opportunities to improve cold resilience, particularly against episodic chilling events and abrupt temperature fluctuations that may become increasingly important under climate change.

## 1. Introduction

Global agriculture is increasingly constrained by climate variability, with cold stress emerging as a critical determinant of crop productivity and geographic distribution.^1^ Chilling (0–15 °C) and freezing (<0 °C) conditions disrupt cellular homeostasis by destabilizing membranes, inhibiting enzymatic activities, and promoting excessive accumulation of reactive oxygen species (ROS), ultimately leading to metabolic dysfunction and yield penalties.^2^ These effects extend beyond immediate physiological damage, as cold stress also suppresses photosynthetic efficiency and carbon assimilation, thereby limiting biomass accumulation across major crops.^3^ Importantly, climate change does not necessarily imply sustained cooling in tropical regions; rather, increasing climatic variability may expose crops to abrupt temperature fluctuations, episodic cold snaps, and recurrent chilling events outside their historical thermal range.^4^ Such events may be particularly consequential for tropical and subtropical crops, which evolved under relatively warm and comparatively stable thermal conditions and generally possess limited capacity for cold acclimation.^5^ Although agronomic practices such as optimized planting dates or plastic mulching can provide partial relief, genetic improvement remains the most sustainable strategy. Consequently, improving cold tolerance and resilience to recurrent temperature fluctuations has become a strategic priority for sustaining agricultural productivity under changing climatic regimes.^6^

Among tropical crops, oil palm (*Elaeis guineensis*) holds exceptional economic importance as the most productive oil-bearing species, contributing a substantial proportion of global vegetable oil supply while utilizing comparatively less land than other oilseed crops.^7^ Despite this advantage, its cultivation remains geographically restricted to equatorial regions, primarily due to its pronounced sensitivity to low temperatures.^8^ Optimal growth is confined to a narrow thermal range (24–32 °C), and even mild chilling conditions can trigger significant physiological and biochemical disturbances.^9^ Cold exposure in oil palm is associated with reduced chlorophyll content, impaired photosystem II efficiency, enhanced lipid peroxidation, and disruption of membrane integrity, collectively reflecting severe oxidative and metabolic stress.^10^ Importantly, cold-induced perturbations in lipid biosynthesis pathways, particularly the downregulation of fatty acid desaturase (FAD) genes, directly compromise oil accumulation and quality, underscoring the dual impact on both yield and industrial value.^2^ Although some genotypic variation in cold responsiveness has been reported, including differential activation of stress-responsive genes, the overall adaptive capacity of oil palm remains limited.^11^ This restricted thermal plasticity represents an important constraint for cultivation in subtropical or marginal environments, where future production may be exposed not to sustained low temperature but to episodic cold snaps, recurrent chilling, and large short term temperature fluctuations.^7^

Substantial progress has been made in elucidating the molecular basis of cold stress responses in model plants, revealing highly coordinated regulatory networks.^2^ Central pathways such as the C-repeat binding factor (CBF), cold-responsive (COR) module, along with calcium signaling, mitogen-activated protein kinase (MAPK) cascades, and hormone-mediated regulation, orchestrate adaptive responses through transcriptional reprogramming, osmotic adjustment, and ROS detoxification.^12^ However, the extent to which these conserved mechanisms operate in oil palm remains insufficiently resolved. Although recent transcriptomic and proteomic studies have identified candidate regulators, including transcription factors and genes associated with antioxidant defense and lipid metabolism, these findings remain largely descriptive and fragmented.^13^ A critical knowledge gap persists: there is no integrated framework linking early molecular signaling events to downstream functional outcomes, particularly in the context of oil biosynthesis and adaptation to recurrent or prolonged chilling.^8^ Furthermore, the translation of molecular insights into breeding applications is constrained by intrinsic challenges, including the long juvenile phase (typically 8–10 years), high heterozygosity, and limited genomic resources of oil palm.^14^ These limitations hinder the efficiency of conventional breeding and necessitate the integration of advanced molecular approaches.^15^ Addressing cold sensitivity in oil palm is therefore not only a biological challenge but also a strategic imperative for maintaining stable oil production as climatic variability increases and episodic cold events become potential constraints in marginal production environments.^14^ A deeper mechanistic understanding of responses to abrupt, recurrent, and prolonged chilling is essential for identifying key regulatory nodes and developing targeted interventions.^16^ Emerging tools such as genome-wide association studies (GWAS), quantitative trait locus (QTL) mapping, and genome editing technologies (e.g., CRISPR-Cas9) provide new opportunities to accelerate genetic improvement, yet their effective application depends on a robust and integrated knowledge framework.^17^ In this context, the present review provides a comprehensive and critical synthesis of cold stress responses in oil palm, encompassing physiological, biochemical, and molecular dimensions. We evaluate recent advances in the identification of key regulatory components and critically assess the potential of modern molecular breeding strategies for enhancing resilience to low-temperature stress. By highlighting existing knowledge gaps and outlining a roadmap for future research, including priorities for multi-omics integration and functional validation of candidate genes, this review aims to support the development of climate-resilient oil palm cultivars capable of maintaining productivity under episodic and recurrent suboptimal temperature conditions associated with increasing climatic variability.

## 2. Physiological and Molecular Underpinnings of Cold Stress Responses in Oil Palm and Oilseed Crops

Cold stress responses in oil palm involve many of the signaling and metabolic components characterized in temperate crops, but the available evidence points to important differences in their timing, amplitude, and coordination.^18^ Rather than indicating the absence of canonical cold-response machinery, current evidence suggests that these regulatory differences may contribute to the comparatively limited acclimation capacity of oil palm.^10^ The following sections therefore examine these processes individually, emphasizing where evidence supports conserved mechanisms, where oil palm appears mechanistically distinct, and where conclusions remain provisional because of limited functional validation.^18^

### 2.1. Cold Perception and Ca^2+^‑Mediated Early Signaling

Cold perception in plants begins with plasma membrane rigidification, leading to rapid Ca^+2^ influx that triggers downstream signaling cascades.^19^ This physical change in membrane fluidity acts as a primary thermosensory mechanism, enabling plants to rapidly convert external temperature fluctuations into intracellular biochemical signals.^20^ As illustrated in Fig. 1, in oil palm, early transcriptomic analyses reveal upregulation of *EgCBL*, *EgCIPK*, CaM-like proteins (CMLs), and calcium-dependent protein kinase (CDPK) isoforms, which act as primary Ca^+2^ sensors under chilling stress.^21^ These Ca^+2^ sensors function as molecular decoders that translate transient Ca^+2^ signatures into specific phosphorylation events, thereby activating downstream signaling pathways.^22^ Additional mechanosensitive channels, such as MCA1 and MCA2, mediate cold-induced Ca^+2^ influx and contribute to both CBF-dependent and independent pathways. The activation of these channels ensures rapid amplification of the calcium signal, which is essential for initiating early stress responses before irreversible cellular damage occurs.^23^ Ca^+2^-regulated transcription factors, including *CAMTA1–3*, are induced under low temperatures and facilitate the expression of cold-responsive genes.^24^ These transcription factors serve as critical intermediates linking calcium signaling to transcriptional reprogramming, particularly through binding to CGCG-box elements in promoter regions of stress-responsive genes.^25^ In *Brassica napus*, *SCREAM/ICE1, NAC29/56, DREB1B, ERF70*, and *TUB6* respond robustly to early Ca^+2^ signaling, promoting downstream transcriptional reprogramming,^26^ while in soybean, tolerant cultivars exhibit elevated *GmCBLs, GmCDPKs*, and *GmCMLs*.^27^ This enhanced responsiveness in temperate and semi-tolerant species reflects a more efficient coupling between Ca^+2^ perception and transcriptional activation, allowing rapid deployment of protective gene networks.^26^

**Figure 1.**
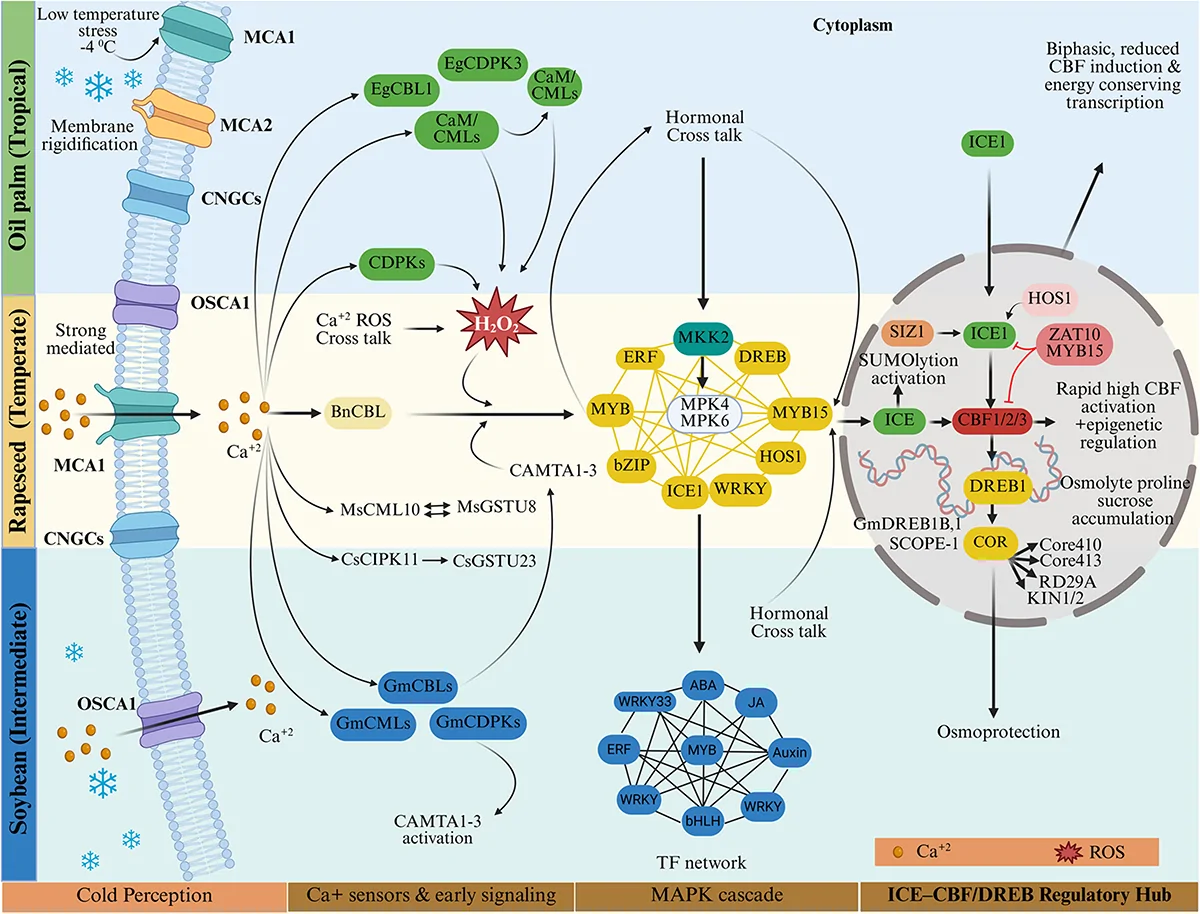
Comparative framework of cold-stress perception, signaling, and transcriptional regulation in oil palm and temperate oilseeds.

Cross-talk between Ca^+2^ and reactive oxygen species (ROS) is exemplified by *MsCML10* interacting with *MsGSTU8*, linking early Ca^+2^ signals to oxidative stress defense,^28^ and *CsCIPK11* phosphorylates *CsGSTU23* to enhance cold tolerance.^29^ This interaction highlights the dual role of ROS as both signaling molecules and damaging agents, where tightly regulated Ca^+2^-ROS interplay ensures controlled signal propagation while minimizing oxidative injury.^28^ Other components include HSFC1, ZAT12, ZF, ZAT10, RAV1, CZF1, and MKK2-mediated MPK4/6 activation observed in plants, integrating cold phosphorylation events.^30,31^ These kinase cascades further amplify the signal and provide multiple regulatory checkpoints for fine-tuning stress responses.^30^ Importantly, oil palm exhibits biphasic induction of CBF1-3, correlated with sucrose and proline accumulation, whereas evidence for comparable *LOS2-*mediated repression has not been clearly established in oil palm. This biphasic pattern suggests a delayed or less synchronized transcriptional activation compared to the rapid and sustained CBF induction observed in cold-tolerant species.^32^ In rapeseed, overexpression of *BnCBL1-2* enhances Ca^+2^ flux, while ICE1-like TFs are strongly induced,^33^ reinforcing the importance of efficient early signaling. Taken together, the available evidence points to differences in signal kinetics and amplification rather than a complete divergence in the components of cold perception. In oil palm, the biphasic induction of CBF genes and associated changes in compatible solutes suggest dynamic activation of the cold-response network, whereas studies in temperate oilseeds provide evidence for more rapid calcium-dependent signal propagation. Whether these differences arise from altered channel activity, calcium-sensor coupling, or downstream kinase activation remains unresolved. Resolving these possibilities will require time-resolved measurements of cytosolic Ca^+2^ dynamics, coupled with functional analysis of candidate signaling components, to determine whether altered signal kinetics contribute causally to cold sensitivity in oil palm.

### 2.2. MAPK Cascades and Transcriptional Reprogramming

Ca^+2^ signaling feeds into MAPK cascades, which amplify stress signals and regulate transcriptional networks.^34^ This integration represents a critical step in signal transduction, where transient calcium signatures are converted into sustained phosphorylation events that can effectively modulate gene expression.^35^ MAPK cascades typically operate through a three-tiered kinase module (MAPKKK–MAPKK–MAPK), enabling signal amplification, specificity, and cross-talk with other signaling pathways^36^ (Fig. 1). In rapeseed, cold exposure induces ERF, bHLH, NAC, MYB, and WRKY TFs, along with stress genes *COR413-PM2, HOS1, ICE1, MYB15*, and *WRKY33*, coordinating gene expression and metabolic adjustments.^37^ These transcription factors function as central regulators that integrate upstream kinase signaling with downstream stress-responsive gene activation.^38^ Soybean exhibits similar integration, where *GmCBLs* and *GmCDPKs* interact with MAPK modules to enhance downstream TF activation.^27,39^ This cooperative interaction between calcium-dependent kinases and MAPK pathways ensures efficient signal propagation and reinforces transcriptional reprogramming under stress conditions.^27^ In oil palm, ABA-linked NAC TFs are induced under cold stress, but the regulatory relationship between their activation and upstream MAPK signaling remains insufficiently resolved.^9^ The MKK2–MPK4/6 cascade has been implicated in cold-responsive signaling and can regulate downstream cold responsive transcriptional programs, including those involving ICE1 and DREB1.^40^ Phosphorylation of these transcription factors not only enhances their stability but also modulates their DNA-binding activity, thereby influencing the expression of a wide range of cold-responsive genes.^41^ Genome-wide rapeseed analyses reveal bZIP, MYB, NAC TFs and numerous DEGs involved in hormone signaling, ribosome function, and carbohydrate metabolism during cold stress.^42,43^ This extensive transcriptional reprogramming reflects a highly dynamic and coordinated response that integrates metabolic, developmental, and stress-related pathways.^40^

Hormonal integration, including ABA, JA, auxin, and brassinosteroids, further intersects with MAPK signaling to coordinate transcriptional and metabolic responses under low temperature.^44^ These interactions provide an additional regulatory layer through which plants balance stress defense with growth and energy expenditure.^40^ In oil palm, the available evidence suggests that NAC- and hormone-associated transcriptional programs participate in cold responses, but the extent to which these programs are directly controlled by MAPK phosphorylation remains unresolved. By contrast, studies in temperate oilseeds have provided stronger evidence linking MAPK modules with downstream transcription factors involved in cold adaptation.^40^ Rather than assigning a single conserved MAPK pathway to oil palm, future work should determine the temporal relationships among Ca^+2^ signaling, MAPK activation, transcription-factor phosphorylation, and downstream gene expression. Time-resolved phosphoproteomic and transcriptomic analyses, combined with functional assays of candidate MAPK–TF interactions, could help distinguish conserved signaling modules from oil-palm-specific regulatory features.

### 2.3. ICE-CBF/dreb Regulatory Hub

The ICE-CBF/DREB module is the central transcriptional hub controlling cold-responsive gene expression across plant species. This module link upstream cold perception and signal transduction to large-scale transcriptional reprogramming^45^ (Fig. 1). ICE1 (Inducer of CBF Expression 1), a MYC-like bHLH transcription factor, binds to promoter regions of CBF/DREB genes and initiates a transcriptional cascade that activates downstream stress-responsive genes.^2^ In oil palm, multiple ICE1 homologs (Unigene615, Unigene5046 and Unigene21287) show biphasic induction under chilling, accompanied by changes in downstream CBF/DREB1 transcripts (eight contigs, including CL2890.Contig1, CL4552.Contig2).^46^ Although some of these CBF/DREB-associated regulatory elements have been reported to contain sequence variation in putative DRE motifs, the functional consequences of these changes for transcription-factor binding and cold responsiveness have not been experimentally established.^45^ Therefore, they should be considered potential regulatory constraints rather than demonstrated causes of reduced CBF activation. The biphasic expression pattern instead indicates that the ICE-CBF/DREB network is dynamically regulated during chilling, with an early response followed by a subsequent adjustment phase.^46^ ICE1 activity is modulated by post-translational regulators, including SIZ1, ZAT10, MYB15, and HOS1, which integrate SUMOylation, redox status, and ubiquitination pathways.^47^ These post-translational modifications are critical for fine-tuning ICE1 stability and activity; for instance, SUMOylation enhances ICE1 stability, whereas ubiquitination mediated by HOS1 promotes its degradation, thereby providing a dynamic balance between activation and repression of cold-responsive transcription.^48^ Downstream cold-responsive (COR) genes, including *COR410/413, RD29A, KIN1/2*, together with the genes involved in proline and sucrose metabolism, contribute to membrane stabilization, osmotic adjustment, and cellular protection.^12,32^ In oil palm, the available evidence therefore supports the presence of a functional cold-responsive transcriptional framework, although the connectivity and regulatory strength of individual components remain incompletely characterized.^18^

In rapeseed, ICE1 and multiple *BnCBF* homologs (*BnCBF1*), together with *DREB1B*, and COR genes (*COR15A/B*) exhibit strong, rapid induction, under cold stress, while epigenetic regulation contributes to the maintenance of stress responsive transcription.^26^ Soybean studies similarly identify *GmDREB1B;1* and *SCOF-1* as a regulator connecting cold-responsive transcription with osmolytes accumulation and ROS detoxification.^49,50^ Ca^+2^-dependent signaling can further converge on ICE1–CBF regulation through CAMTA proteins and upstream membrane-associated signaling components.^51,52^ These comparative studies illustrate how the ICE-CBF/DREB module can be coupled to rapid signal perception and sustained downstream protection, but they do not by themselves establish that the same regulatory architecture operates with equivalent kinetics or strength in oil palm.^46^ A key unresolved issue is how activation and repression are balanced within the oil palm ICE–CBF/DREB network. Although LOS2-mediated repression has not been demonstrated in oil palm, it remains unclear whether this reflects the absence of an identifiable LOS2 homolog, incomplete genome annotation, or insufficient functional characterization.^32^ Thus, the major knowledge gap concerns how efficiently conserved cold-response components are coordinated and sustained during chilling. Addressing this will require promoter analyses, time-resolved transcriptomics, and functional validation of ICE–CBF/DREB regulators.^53^

The figure summarizes four major stages of the cold-response pathway: cold perception, Ca^2+^ sensing and early signaling, MAPK-mediated signal transduction, and the ICE–CBF/DREB regulatory hub. In oil palm, low-temperature-induced membrane rigidification is associated with Ca^2+^ influx and activation of Ca^+2^-responsive components, including CBL/CIPK, CaM/CML, and CDPK modules, followed by Ca^2+^–ROS interactions and downstream transcriptional regulation. Rapeseed and soybean are presented as comparative systems illustrating conserved Ca^+2^-dependent signaling, MAPK activation, transcription-factor networks, hormonal integration, and downstream osmoprotective responses. The ICE–CBF/DREB module represents the major transcriptional interface linking upstream signaling to cold-responsive genes involved in osmoprotection and stress adaptation. The figure emphasizes conserved regulatory architecture while highlighting differences in the reported dynamics and coordination of cold responses among species. Molecular components shown in the figure are based on evidence from oil palm and comparative studies in temperate oilseeds; their presence in the network does not necessarily imply direct functional validation in oil palm.

### 2.4. Membrane Lipid Remodeling and Regulatory RNAs

Cold stress affects membrane fluidity and stability, necessitating rapid lipid remodeling.^54^ This process is essential because low temperatures reduce membrane flexibility, leading to phase transitions that can impair membrane-bound proteins and transport systems.^55^ To counteract this, plants actively adjust lipid composition, particularly by increasing the proportion of unsaturated fatty acids to maintain optimal membrane functionality.^56^ In rapeseed and *Olea europaea*, cold exposure increases the expression of fatty acid desaturases *FAD2*, *FAD8*, and stearoyl-ACP desaturases (SAD), enhancing unsaturation and membrane fluidity.^57^ These enzymes introduce double bonds into fatty acid chains, preventing tight lipid packing and thereby preserving membrane permeability and protein activity under chilling conditions.^7^ Oil palm, comparatively limited induction of these desaturases has been reported, while cold-responsive regulatory RNAs, including miR156, miR319, have been associated with the regulation of fatty acid metabolism, transcription factor, antioxidants responses, and osmotic adjustment.^58^ These observations suggest that post-translational regulation may contribute to the coordination of lipid metabolism and stress responses, although the functional roles of individual miRNAs remain to be established.^18^

Flavonoids such as vitexin and isovitexin accumulate under cold stress in oil palm, potentially contributing to ROS scavenging and membrane protection. These secondary metabolites not only neutralize reactive oxygen species but also interact with lipid bilayers to stabilize membrane structure, reducing oxidative damage and leakage.^10^ Rapeseed integrates lipid-associated antioxidant mechanism, including BnCML and BnCAT, which contribute to maintaining membrane integrity under freezing conditions.^59^ Such antioxidant systems complement lipid remodeling by protecting membrane components from peroxidation. Further, metabolomic analyses reveal coordination between lipid remodeling and primary metabolites, such as sugars and amino acids, supporting osmotic balance.^56^ These metabolites act as compatible solutes, stabilizing cellular structures and maintaining turgor pressure under stress conditions.^60^ Rather than indicating a simple shift from enzymatic lipid desaturation to miRNA-mediated regulation, the available evidence suggests that membrane protection under cold stress involves coordinated lipid, regulatory, antioxidant, and metabolic processes.^61^ In oil palm, however, the relative contributions of fatty acid desaturation, regulatory RNAs, and secondary metabolites remain insufficiently resolved.^18^ Integrated lipidomic and small RNA profiling, together with functional validation, will therefore be important for determining how these mechanisms collectively influence membrane stability during chilling.^61^

### 2.5. Cold-Induced Osmotic Stress and Water Transport Limitation

Cold stress significantly impairs water uptake, leading to osmotic stress and reduced hydraulic conductivity.^62^ This occurs because low temperatures increase water viscosity, decrease root permeability, and induce partial cellular dehydration, collectively limiting turgor maintenance.^63^ In oil palm, cold stress triggers transcriptional reprogramming, including genes involved in water transport, while evidence from other plant species indicates that PIP1- and PIP2-type aquaporins, such as the putative oil-palm homologs *EgPIP1;1 and EgPIP2;1*, are downregulated under chilling.^64,65^ However, whether these expression changes directly reduce aquaporin activity or root hydraulic conductivity in oil palm remains unresolved, because functional characterization of these proteins under cold stress is lacking. Consequently, water movement within the plant may be constrained under chilling, potentially exacerbating osmotic stress.^65^ Correspondingly, qPCR analyses reveal that *EgCBF1/2/3* induction is accompanied by the upregulation of osmolyte biosynthesis genes, including *P5CS* (proline biosynthesis) and sucrose metabolism enzymes *SUSY* and *SPS*, resulting in enhanced accumulation of compatible solutes such as proline and sucrose, which contribute to osmotic adjustment and may help alleviate cellular dehydration under cold stress.^66–68^ The proposed role of LOS2 in this process should therefore be interpreted cautiously, as the available evidence does not establish either definitive genomic absence of an LOS2 homolog or a functional contribution of LOS2-mediated repression in oil palm.^69^

In soybean, cold-tolerant cultivars exhibit coordinated osmoprotection through upregulation of *TPS/TPS13* (trehalose synthesis), *GmP5CS1/2* (proline biosynthesis), and *GmSPS8/18* (sucrose phosphate synthase) under ICE1-mediated regulation.^70,71^ These metabolites act as compatible solutes, stabilizing membranes, maintaining cytoplasmic osmolarity, and protecting proteins from cold-induced denaturation.^71^ Genetic mapping studies have identified multiple quantitative trait loci (QTLs associated with cold germination and osmotic stability across several chromosomes, reflecting the polygenic nature of osmotic stress resilience.^72,73^ Soybean also maintains aquaporin activity (*PIP2;5*), allowing sustained water transport, while GmCIPK5-GmCBL1 complexes mediate ABA-driven stomatal and osmotic closure, integrating hydraulic control with hormonal signaling.^74,75^ Rapeseed responds to cold via ERD4 family osmolyte stabilization and metabolic accumulation of amino acids, sugars, and sugar alcohols, as revealed by metabolome analyses.^76^ These metabolites not only balance osmotic pressure but also act as ROS scavengers, supporting both osmotic and oxidative stress protection.^77^ Conserved cold-responsive (COR) genes such as *RD29A* have been widely reported to function in osmotic adjustment and stress protection in plants.^78^ In oil palm, COR-like homologs, including putative *EgCOR410*, are predicted from genome annotations; however, sequence similarity and cold-responsive expression alone do not establish a direct role in osmotic protection. Functional validation through gene perturbation, protein-level analysis, or complementation assays is therefore needed to determine whether *EgCOR410* contributes causally to cold adaptation.^32^ Consistent with this regulatory framework, increased proline levels are positively associated with COR gene expression, providing correlative evidence for their potential involvement in osmotic protection under cold stress.^79,80^ Rather than attributing osmotic limitation directly to individual PIP or COR homologs, the available evidence supports a model in which cold-induced changes in water transport and compatible-solute metabolism jointly influence osmotic adjustment, while the specific contributions of individual candidate genes remain to be established.^78^These comparative mechanisms of membrane lipid remodeling, osmotic adjustment, and water transport under cold stress are summarized in Fig. 2.

**Figure 2.**
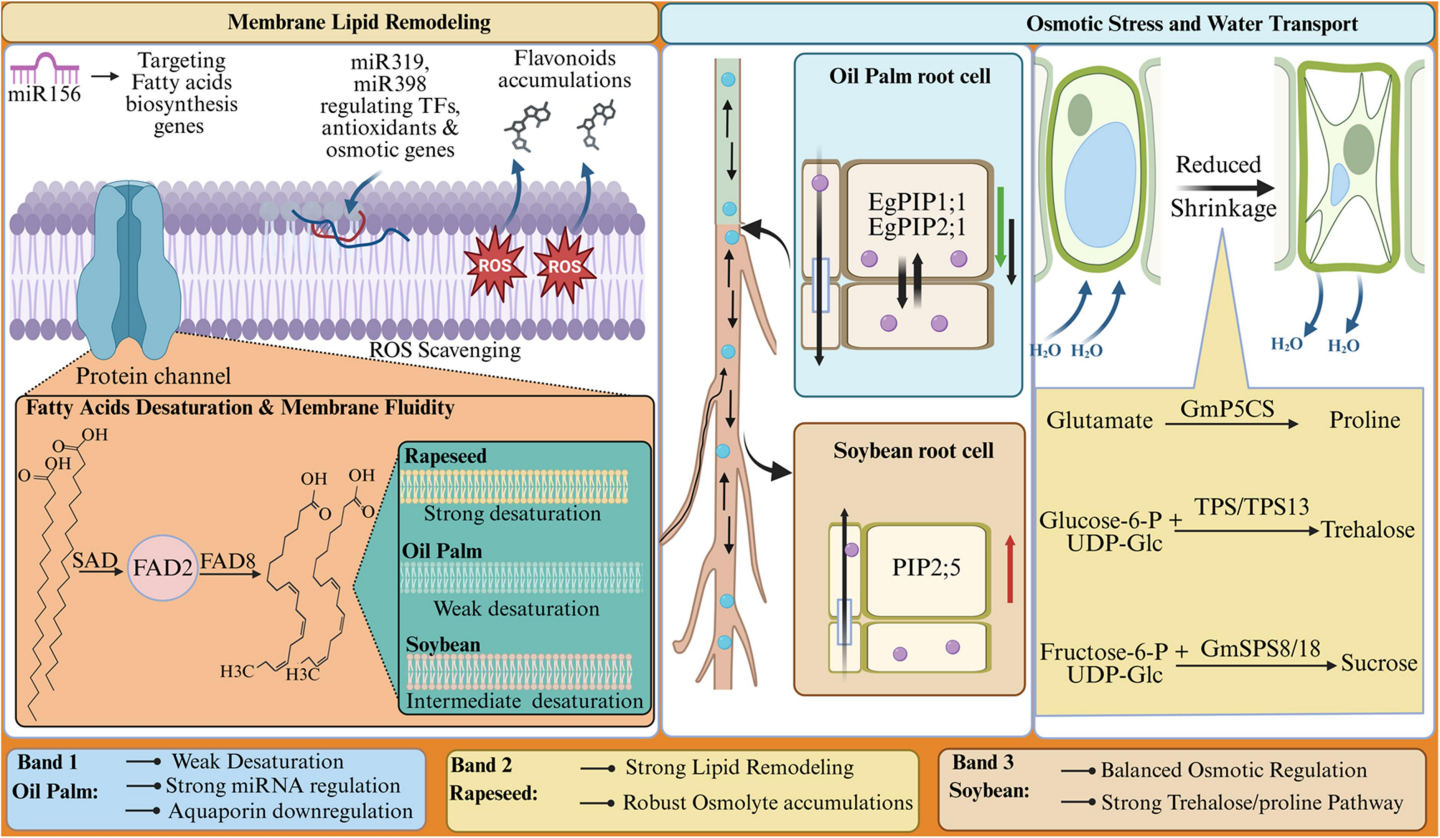
Comparative molecular mechanisms of cold stress tolerance and adaptive responses in oil palm, rapeseed, and soybean. The illustration highlights three primary regulatory axes: (left) membrane lipid remodeling and fatty acid desaturation pathways. miR156, miR319, and miR398 regulate fatty acid biosynthesis genes and transcription factors (TFs) to manage ROS scavenging and membrane fluidity through the SAD-FAD2-FAD8 enzymatic cascade. (center) Comparative osmotic stress and water transport dynamics in root cells. Oil palm shows distinct aquaporin downregulation (*EgPIP1;1, EgPIP2;1*) compared to soybean (*PIP2;5*), impacting xylem water flux. (right) osmotic regulation and cellular homeostasis. The diagram details the metabolic biosynthesis of proline (via *GmP5CS*), trehalose (via *TPS/TPS13*), and sucrose (via *GmSPS8/18*) to mitigate cell shrinkage. Summary bands (bottom) categorize the species-specific strategies: weak desaturation and aquaporin downregulation in oil palm (band 1); robust lipid remodeling in rapeseed (band 2); and balanced osmotic regulation in soybean (band 3).

### 2.6. Oxidative Stress and Antioxidant Defense

Cold stress induces ROS overproduction due to impaired photosynthesis, mitochondrial electron transport disruption, and metabolic imbalance, necessitating robust antioxidant systems to prevent cellular damage.^81^ In oil palm, SOD, CAT, and POD activities rise transiently under chilling, accompanied by induction of *COR410/413* and regulation by miR156 and miR395.^32,61^ These antioxidants provide initial detoxification of superoxide radicals and hydrogen peroxide, but persistent lipid peroxidation, indicated by elevated malondialdehyde (MDA) levels, suggests that antioxidant activation may not fully prevent oxidative damage under prolonged chilling.^61^ *EgCBF1/3* positively correlates with SOD activity, linking transcriptional cold response with ROS management, while electrolyte leakage inversely correlates with *EgCOR410*, indicating an association between cold-responsive gene expression and membrane integrity.^32^ The temporal dynamics of these responses are therefore important for understanding how effectively oil palm maintains redox homeostasis during prolonged stress.^61^ Comparative studies provide useful context for interpreting this response. In rapeseed, APX, GPX, GSTs, BnCML, BnCAT, and BnNIR contribute to a multi-tiered antioxidant network that supports ROS detoxification and redox homeostasis under freezing stress.^59^ ROS responsive regulators such as *ZAT12* are also strongly induced, illustrating the integration of ROS sensing with transcriptional regulation.^82^The contrasting cold-induced ROS accumulation and antioxidant defense mechanisms in oil palm and rapeseed are summarized in Fig. 3.

**Figure 3.**
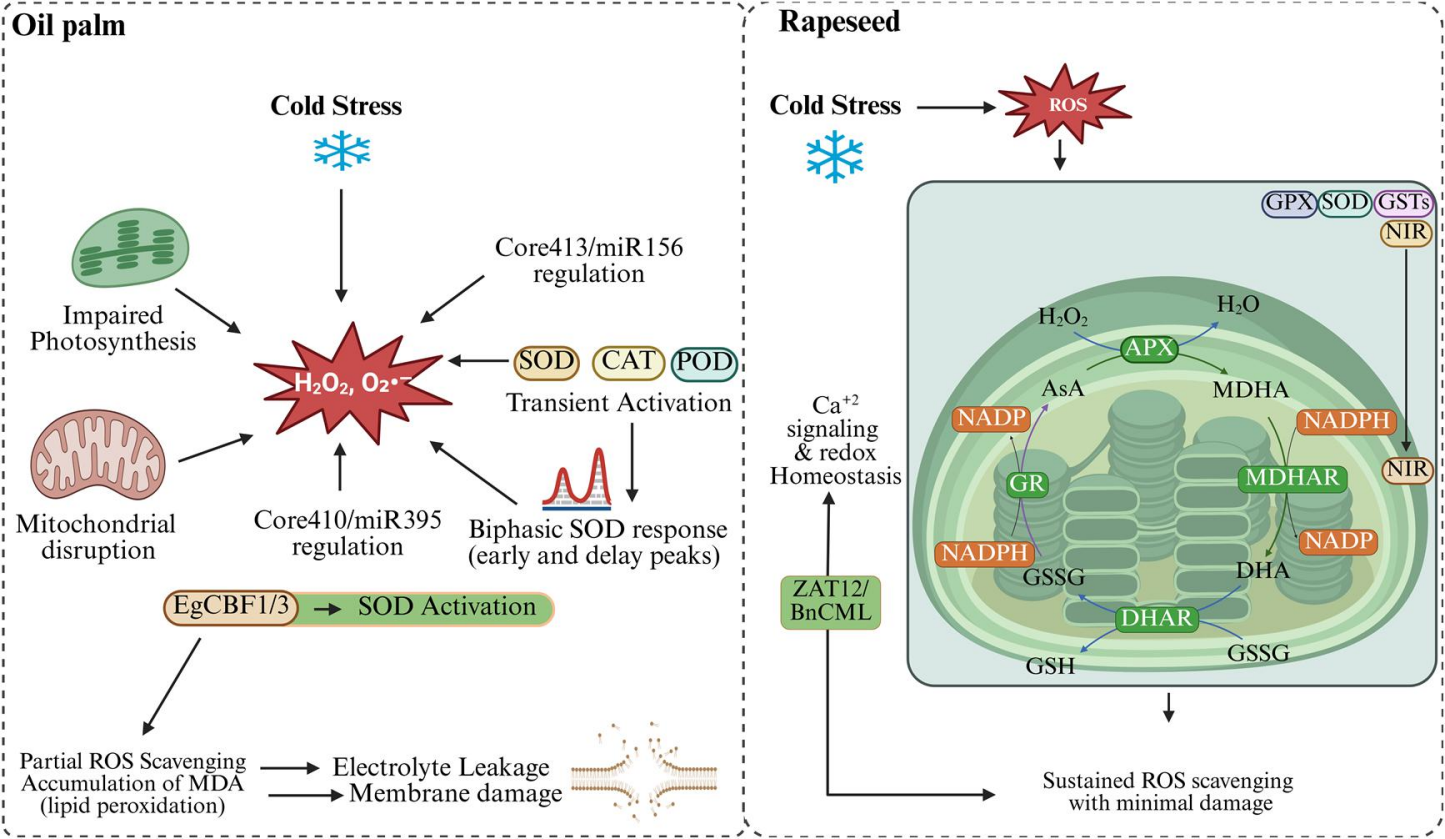
Comparative analysis of oxidative stress management and antioxidant defense mechanisms in oil palm (*Elaeis guineensis*) and rapeseed (*Brassica napus*) under cold stress. The left panel illustrates the cold-sensitive response of oil palm, characterized by ROS overproduction (H_2_O_2_ and O_2_•^−^) stemming from mitochondrial disruption and impaired photosynthesis. Transient activation of antioxidant enzymes (sod, cat, pod) and transcriptional regulators such as *EgCBF1/3* and miRnas (miR156, miR395) fail to achieve complete ROS detoxification, leading to MDA accumulation, electrolyte leakage, and membrane damage. The right panel depicts the cold-tolerant response in rapeseed, featuring a robust antioxidant network including the glutathione–ascorbate (AsA–GSH) cycle, sustained enzyme activity (GPX, GSTs, NIR), and Ca^+2^-mediated redox homeostasis regulated by ZAT12 and BnCML, resulting in efficient ROS scavenging and minimal cellular damage.

Proteomic and metabolomic analyses further reveal biphasic SOD responses in oil palm with peak at 4 hours and 7 days, indicating temporally distinct phases of antioxidant activation.^32,83^ Genome-wide studies in rapeseed have identified more than 5,669 cold-responsive DEGs affecting antioxidant enzymes, redox buffers, and signaling components.^42^ Together, these findings highlight the importance of temporal regulation and coordination among ROS production, antioxidant capacity, and transcriptional responses in determining cold-induced cellular damage.

### 2.7. Integrated Cold Response Pathways and Hormonal Regulation

Hormonal signaling orchestrates systemic cold adaptation by coordinating transcriptional, metabolic, and osmotic responses to optimize survival under low temperatures.^84^ In rapeseed, transcriptome analyses under freezing stress revealed extensive reprogramming of hormone signaling pathways, including ABA signaling components (PYR/PYL receptors and SnRK2 kinases), ethylene regulators (EIN3/EIL), and jasmonic acid-related genes. These changes were part of a broader hormone-enriched transcriptional response involving numerous differentially expressed genes under freezing conditions.^85,86^ These hormones collectively modulate stress-responsive transcription factors, antioxidant defenses, osmolyte accumulation, and metabolic reprogramming, contributing to coordinated stress adaptation.

In oil palm, cold induces shifts in ABA and GA levels, along with upregulation of *EgCBF1/2/3*, linking hormone balance with transcriptional and osmotic responses.^9^ ABA has been implicated in the regulation of cold responsive pathways, whereas reduced GA signaling may contribute to the suppression of growth-related processes under stress.^86^ Multi-hormone crosstalk, involving AUX/IAA, JA, brassinosteroids (BR), and MAPK pathways may further integrate growth-defense trade-offs, and resource allocation between survival and development.^87^ In soybean, GmCIPK2-GmCBL1-mediated ABA signaling enhances stomatal closure and osmolyte accumulation, reducing water loss and promoting cellular homeostasis.^88^ In rapeseed, KEGG analyses highlight ABA, JA, and ethylene signaling as a major cold-responsive pathways, indicating extensive integration.^89^ Additional regulatory layers include MKK2-mediated TF activation, ABA-mediated modulation of ICE1, and repression of GA-dependent growth pathways.^59,89^ Comparatively, oil palm appears to integrate ABA-, GA-, JA-, auxin-, and BR-associated signals with cold-responsive transcription and metabolic adjustment, rather than relying on a single hormone pathway.^18^ The key distinction from temperate oilseeds may therefore lie less in the presence of individual hormone-responsive components than in how these signals are temporally coordinated with calcium signaling, MAPK activity, transcriptional reprogramming, and resource allocation.^87^ This integrated hormonal–metabolic framework provides a useful basis for understanding how oil palm balances stress protection with continued growth under episodic chilling.

## 3. Natural Variation and Genetic Resources for Cold Resilience in Oil Palm and Oilseed Crops

### 3.1. Diversity in Germplasm

Natural genetic variation represents a fundamental resource for improving cold tolerance in oil palm, particularly given the species’ narrow ecological adaptability and long breeding cycle.^11^ Comparative studies between *E. guineensis* and *E. oleifera* have revealed significant differences in stress resilience, with *E. oleifera* exhibiting relatively higher tolerance to abiotic stresses, including low temperature, likely due to its broader genetic base and adaptive evolution in more variable environments.^90^ Transcriptomic analyses comparing these species have identified differential expression of stress-responsive genes, including transcription factors such as CBF/DREB, NAC, and WRKY, as well as antioxidant-related genes such as SOD and APX, suggesting that inherent genetic variation underlies differences in stress tolerance mechanisms.^91^ In interspecific hybrids (*E. guineensis* × *E. oleifera*), improved stress resilience has been associated with enhanced expression of lipid metabolism genes such as *FAD2* (Fatty acid desaturase 2) and *SAD* (Stearoyl-ACP desaturase), which contribute to increased membrane fluidity under cold conditions.^92^ In addition, variation in oil biosynthesis regulators such as WRI1 (WRINKLED1) and DGAT (Diacylglycerol acyltransferase) has been linked to both stress response and oil accumulation, indicating a functional overlap between metabolic and stress adaptation pathways.^7^

Within *E. guineensis*, significant diversity exists among landraces and breeding populations, with certain genotypes exhibiting enhanced tolerance to suboptimal temperatures.^93^ However, detailed functional characterization of cold tolerance at the genotype level remains limited. Studies in plants have shown that osmotic adjustment is commonly associated with the induction of *P5CS* and sugar metabolism genes such as *SUSY* and *TPS*, together with enhanced antioxidant defense involving CAT, POD, and GST gene families, suggesting that similar mechanisms may contribute to stress adaptation in oil palm.^94,95^ Recent RNA-seq studies have further revealed genotype-specific expression of stress-responsive transcription factors, including EgMYB, EgNAC, and EgWRKY, highlighting the role of transcriptional plasticity in cold adaptation.^91,96^ Comparative studies in other oil crops, such as rapeseed (*Brassica napus*), have identified substantial natural variation in cold tolerance associated with differential expression of *BnCBF* genes and downstream COR targets, reinforcing the importance of transcriptional regulation in stress resilience.^42^ Similarly, in olive (*Olea europaea*), genotypic variation in cold tolerance has been linked to differential expression of lipid desaturation genes such as *FAD8*, which modulate membrane stability under low temperatures.^57^ Despite these advances, the genetic diversity of oil palm for cold tolerance remains underexplored, and systematic evaluation of germplasm resources is required to identify elite genotypes for breeding programs.^18^ A representative summary of germplasm resources and their associated stress-responsive genes is presented in Table 1.

**Table 1.** Natural genetic variation and germplasm Resources for cold tolerance.

Crop / Species	Germplasm Type	Observed Cold Tolerance	Key Stress-Responsive Genes	Functional Notes	References
*Elaeis guineensis*	Landraces & breeding populations	Moderate	*EgCBF, EgICE1, EgMYB, EgNAC, EgWRKY*	Transcriptional regulation of osmolytes accumulation, antioxidants defense, and stress-responsive pathways	^7^
*Elaeis oleifera*	Wild populations	High	*EoCBF, EoFAD2, EoSAD, EoWRI1*	Regulation of fatty acids composition, membrane fluidity, antioxidant protection, and lipid metabolism	^7^
Inter-specific hybrid (*E. guineensis × E. oleifera*)	Experimental lines	Enhanced	*EgCBF, EgFAD2, EgDGAT*	Integration of stress-responsive signaling with membrane lipid remodeling and enhanced lipid biosynthesis	^97^
Rapeseed (*Brassica napus*)	Diverse cultivars	Variable	*BnCBF, BnCOR*	CBF-COR-mediated cold acclimation, membrane stabilization, osmotic adjustment, and antioxidant defense	^59^
Soybean (*Glycine max*)	Cultivars & landraces	Moderate	*GmDREB1, GmCBF, GmCOR*	DREB/CBF-COR-mediated transcriptional regulation, osmoprotection, and antioxidant responses	^98^

### 3.2. Identification of QTLs and Candidate Genes

The identification of quantitative trait loci (QTLs) and candidate genes associated with cold tolerance is critical for accelerating genetic improvement in oil palm.^11^ Recent genome-wide association studies (GWAS) and transcriptomic analyses have begun to uncover genomic regions linked to stress responses, although studies specifically targeting cold tolerance in oil palm remain limited.^90^ Transcriptome profiling under chilling stress has identified key candidate genes, including *EgCBF*, *EgICE1*, *EgLEA* (Late embryogenesis abundant proteins), and *EgHSP* (Heat shock proteins), which are involved in stress signaling, protein stabilization, and cellular protection.^9^ In addition, genes related to antioxidant defense, such as *EgSOD*, *EgAPX*, and *EgGST*, and osmolyte biosynthesis genes such as *EgP5CS* and *EgTPS*, have been consistently associated with cold stress responses, suggesting their potential as molecular markers for breeding.^21^

In oil palm, recent high-throughput sequencing studies have also identified differentially expressed genes involved in hormonal signaling pathways, including *PYR/PYL* (ABA receptors) and *SnRK2* (ABA signaling kinases), indicating the importance of hormone-mediated regulation in cold adaptation.^9^ Furthermore, candidate genes related to lipid metabolism, such as *FAD2*, *KAS* (Ketoacyl-ACP synthase), and *DGAT*, have been implicated in maintaining membrane integrity and oil biosynthesis under stress conditions.^7^ Comparative genomics approaches have revealed conserved QTL regions associated with cold tolerance in related oil crops. For example, in rapeseed, multiple QTLs linked to cold tolerance have been mapped to genomic regions containing CBF gene clusters, as well as genes involved in carbohydrate metabolism and ROS scavenging.^99^ In soybean (*Glycine max*), GWAS studies have identified loci associated with cold tolerance that harbor genes such as *GmDREB1*, *GmCBF*, and *GmCOR*, which regulate stress-responsive pathways.^100,101^ Similarly, in sunflower (*Helianthus annuus*), QTLs associated with chilling tolerance have been linked to genes controlling membrane stability, antioxidant defense, and hormonal regulation.^102^

Recent advances in multi-omics integration, combining transcriptomics, metabolomics, and proteomics, have further improved the identification of key regulatory networks underlying cold tolerance.^103^ In oil palm, integrated analyses have revealed coordinated regulation of transcription factors (MYB, NAC, WRKY), signaling components (MAPKs, CDPKs), and metabolic genes involved in osmoprotection and lipid metabolism.^21^ Despite these findings, several critical bottlenecks remain. First, the translation of candidate genes and QTLs into practical breeding tools is hindered by the complex genetic architecture of cold tolerance, which likely involves many small-effect loci rather than a few major-effect genes.^102^ Second, the long generation cycle of oil palm (typically 4 to 5 years) makes validation of QTL effects through conventional genetic populations extremely time-consuming.^11^ Third, and most importantly, no candidate gene identified from transcriptomic studies in oil palm has yet been functionally validated (e.g., through heterologous expression, virus-induced gene silencing, or CRISPR-Cas9 editing), leaving their causal roles in cold tolerance unconfirmed.^104^ Therefore, the development of high-resolution genetic maps, functional validation of candidate genes, and incorporation of molecular markers into breeding programs are essential for harnessing natural variation and improving cold resilience in oil palm.^18^

## 4. Challenges and Future Perspectives for Cold Resilience in Oil Palm

### 4.1. Major Challenges in Understanding and Enhancing Cold Tolerance

Despite substantial progress in elucidating plant responses to low-temperature stress, the molecular basis of cold tolerance in oil palm remains incompletely understood, thereby limiting the efficiency of breeding strategies.^21^ These challenges can be broadly categorized into three interrelated domains: molecular knowledge gaps, intrinsic biological constraints, and technical bottlenecks.

#### 4.1.1. Molecular Knowledge Gaps

A key challenge lies in clarifying the early events of cold perception and signal initiation in tropical perennials.^105^ In model plants, components such as COLD1, OSCA1, and CNGCs (cyclic nucleotide-gated channels) facilitate rapid temperature sensing through Ca^2+^ influx.^106^ Extending these findings to oil palm, particularly in defining the role and regulation of *EgCOLD1*, will be crucial for understanding upstream signaling dynamics.^107^ Calcium-dependent signaling networks, including CBL–CIPK modules, are known to participate in chilling responses; however, their integration with upstream sensors and downstream transcriptional regulation requires further resolution.^108^ In addition, coordinated root-to-shoot communication under cold stress represents an important but less-developed area, especially considering evidence from *Arabidopsis* where mobile peptides (e.g., CLE25) and ABA biosynthesis via NCED3 mediate systemic signaling.^109,110^ In oil palm, deciphering how these long-distance signals regulate frond and root responses could provide new insights into whole-plant adaptation. Epigenetic regulation, including DNA methylation and histone modifications, has been shown to control key cold-responsive genes such as CBF, COR, and ICE1 in temperate species, and its integration into oil palm stress biology represents a promising research direction.^111^ However, oil palm-specific epigenetic studies under cold stress are entirely lacking, whether cold-induced changes in DNA methylation at EgCBF loci contribute to the weak transcriptional response observed remains unknown.^107^

#### 4.1.2. Biological Constraints

Molecular challenges are further influenced by intrinsic biological constraints of oil palm as a crop. Although oil palm can reach first harvest approximately 4 to 5 years after planting under favorable conditions, this interval should not be equated entirely with the juvenile phase, and the time required for genetic evaluation varies with the trait, population, and experimental design. For cold-tolerance traits that require mature-plant assessment or multi-year phenotyping under variable field conditions, QTL validation and subsequent breeding cycles may extend over a decade or longer, substantially slowing genetic improvement.^11^ The high heterozygosity of oil palm further complicates genetic analyses, as segregating populations exhibit substantial genetic noise that can mask small-effect QTLs for cold tolerance.^14^ This challenge is compounded by the large stature of mature oil palms (often exceeding 10 m), which limits the use of standard controlled-environment growth chambers and often necessitates field-based evaluation under naturally fluctuating temperatures, where cold events are episodic and difficult to replicate systematically.^11^

#### 4.1.3. Technical Bottlenecks

Functional validation in oil palm is constrained by low transformation efficiency. Although Agrobacterium-mediated transformation systems are available, reported efficiencies (1–5%),^112^ are considerably lower than in model or temperate crops. Together with the 2–3 years required for plant regeneration; this limitation has significantly hindered functional characterization of candidate genes.^14^ Notably, cold-responsive candidates identified through transcriptomic analyses have not yet been extensively validated through stable transgenic or genome-editing approaches in oil palm, in contrast to rapeseed and soybean, where functional are more advanced.^18^

A key priority is therefore to establish a tiered functional-validation pipeline that distinguishes candidate identification from demonstrated gene function. For putative homologs such as *EgPIP1;1, EgPIP2;1*, and *EgCOR410*, comparative sequence and promoter analyses can first establish conservation of functional domains and regulatory elements, followed by time-resolved expression profiling, protein localization, and protein–protein interaction assays under chilling conditions.^64,65^ Candidate genes showing consistent molecular and physiological associations can then be tested using transient expression, protoplast-based assays, heterologous complementation, or model-plant systems, providing functional evidence without requiring immediate stable transformation of oil palm.^11^ The most robust candidates can subsequently be prioritized for targeted gene perturbation, genome editing, or allele-specific validation in oil palm. Integrating these approaches with quantitative measurements of hydraulic conductivity, membrane stability, osmolyte accumulation, and ROS homeostasis would help distinguish genes that are merely cold-responsive from those that causally contribute to cold tolerance.^107^

This limitation is further exacerbated by the scarcity of integrated genomic and phenotypic datasets for cold tolerance. Most transcriptomic studies are conducted on seedlings under controlled conditions, and their relevance to mature palm performance under field conditions remains unclear.^113^ The lack of standardized cold phenotyping protocols hampers cross-study comparisons, while the absence of high-density genetic maps for cold tolerance traits continues to constrain QTL discovery.^11^ Developing standardized phenotyping pipelines and linking physiological traits with genomic, transcriptomic, and functional datasets would therefore provide a stronger basis for prioritizing candidates for validation and breeding.^18^

### 4.2. Future Perspectives and Strategic Opportunities

Emerging technologies provide important opportunities to address these limitations, but their practical deployment in oil palm must be aligned with the crop’s long generation time stature, and transformation constraints.^113^ Accordingly, near-term progress should prioritize high-throughput phenotyping, genomic selection, marker-assisted breeding, exploitation of natural genetic variation, and non-stable functional validation, while genome editing should be considered a longer-term strategy as transformation and regeneration efficiencies improve.^104^ Below, we outline the most promising approaches and their specific applicability to oil palm, as summarized in Fig. 4.

**Figure 4.**
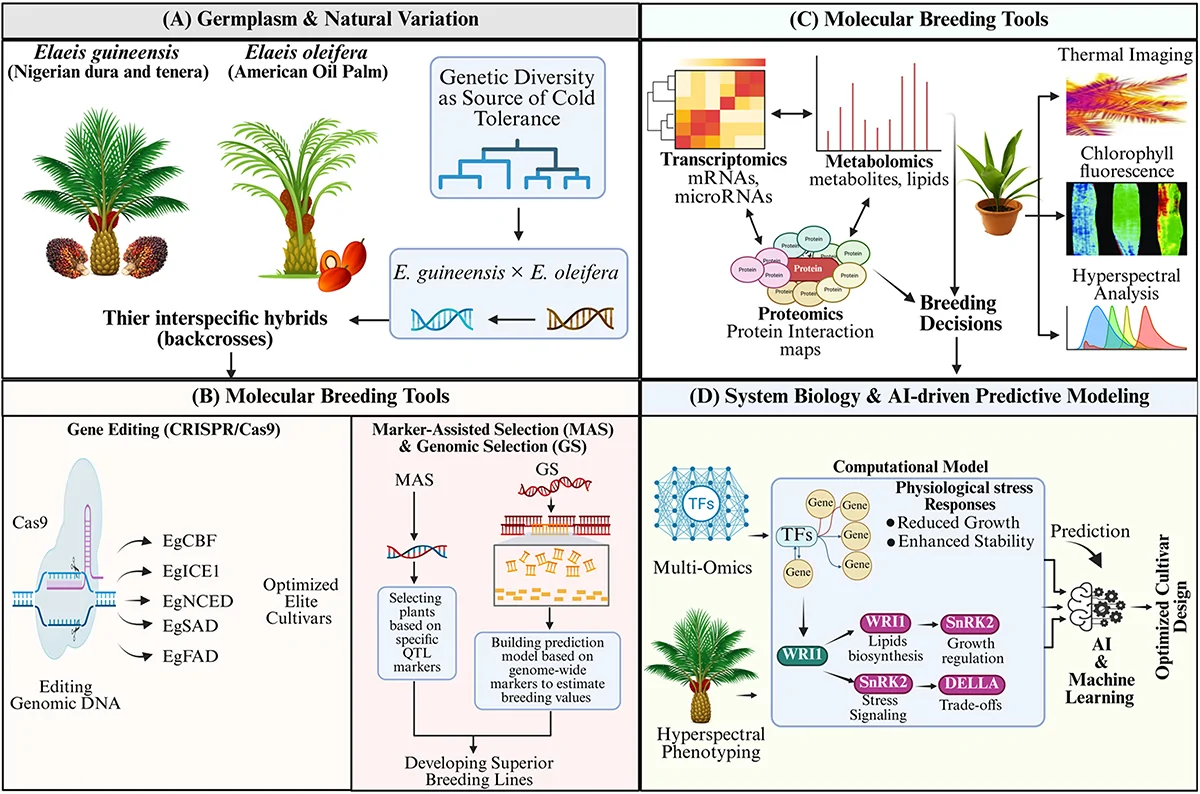
Future prospects for enhancing cold resilience in oil palm (*Elaeis guineensis*) through integrative genetic and molecular approaches.

#### 4.2.1. Genome Editing for Targeted Trait Improvement

Genome editing tools such as CRISPR/Cas9 offer a promising longer-term strategy for targeted modification of cold-responsive genes, including *CBF*/*DREB*, *ICE1*, *NCED*, and lipid metabolism-related genes such as *FAD2*, *FAD8*, and *SAD*, which regulate stress signaling, ABA pathways, and membrane fluidity.^114^ Importantly, CRISPR/Cas9 editing has already been demonstrated experimentally in *E. guineensis*, with successful targeting of *EgPDS* and *EgBRI1* in oil palm protoplasts and regenerated shoots.^115^ Multiplex CRISPR/Cas9 has subsequently enabled targeted editing of *EgFAD2* and *EgPAT*, demonstrating the feasibility of modifying lipid-related traits in oil palm.^116^ More recently, a DNA-free CRISPR/Cas9 ribonucleoprotein system achieved efficient targeted mutagenesis in oil-palm protoplasts, further expanding the potential for transgene-free genome editing.^117^ Studies in model and oilseed crops also indicate that strategic modulation of cold-responsive and lipid-associated pathways can enhance chilling tolerance through activation of downstream protective mechanisms.^118^ For oil palm, promoter editing of *EgCBF* genes could eventually be explored to enhance cold-inducible expression, rather than constitutive activating, potentially minimizing growth-defense trade-offs.^119^ Similarly, targeted modulation of ABA signaling components, including PP2C phosphatases and SnRK2 kinases, may provide opportunities to fine-tune stress responsiveness.^120^ Nevertheless, challenges in stable transformation, regeneration, and efficient recovery of edited plants remain important constraints. Thus, functional characterization through transient expression, protoplast-based assays, heterologous complementation, and physiological validation should continue to support candidate-gene prioritization before cold-tolerance editing is pursued in oil palm.^116^

#### 4.2.2. High-Throughput Phenotyping and Controlled Environment Solutions

In the near term, high-throughput phenotyping offers a practical route for identifying cold-resilient oil palm germplasm.^18^ RGB cameras, thermal infrared cameras, chlorophyll fluorescence imaging, and multispectral/hyperspectral sensors can provide rapid, nondestructive assessment of canopy traits, leaf temperature, photosynthetic performance, and physiological changes.^121^ Seedling-based platforms could enable large germplasm collections to be screened under controlled chilling conditions, followed by field validation of selected candidates.^18^ Integrating imaging-derived traits with measurements of photosynthesis, electrolyte leakage, membrane stability, water status, and antioxidant capacity could improve screening accuracy. Environmental sensors for temperature, humidity, soil moisture, and light intensity can further link plant responses to microenvironmental conditions.^122^ Digital twin technology could provide an emerging nondestructive framework for continuous monitoring of cold responses by integrating sensor, imaging, physiological, and environmental data. Such systems could enable early detection, prediction of genotype-specific responses, and precision phenotyping.^123^ Developing portable, low-cost phenotyping systems suitable for remote oil palm-growing regions should therefore be prioritized.

#### 4.2.3. Multi-Omics Integration and Systems Biology

When integrated with multi-omics datasets (genomics, transcriptomics, proteomics, and metabolomics), these platforms support a systems-level understanding of regulatory networks, including MYB, NAC, and WRKY transcription factors, MAPK/CDPK signaling cascades, and metabolic pathways associated with osmoprotection and lipid remodeling.^124^ In parallel, genome-wide association studies (GWAS), marker-assisted selection, and genomic selection offer potentially more immediately deployable strategies for identifying and accumulating favorable alleles associated with cold tolerance. These approaches have been successfully applied in crops such as soybean, rapeseed, and sunflower,^125–127^ and do not require stable transformation, making them particularly attractive for a recalcitrant perennial crop such as oil palm. Combining genomic prediction with high-throughput phenotyping could enable early selection of promising genotypes before the onset of the long juvenile phase.^128^

#### 4.2.4. An Integrated Molecular Design Breeding Framework

To accelerate genetic improvement, an integrated molecular design breeding framework is required, combining genetic, physiological, and computational approaches.^15^ This strategy should emphasize functional validation of central regulatory genes (*EgCBF*, *EgICE1*, *EgNCED*, *Eg SAD* and *EgFAD*) and their coordinated roles in stress adaptation, while integrating marker-assisted selection and genomic selection as near-term breeding approaches and reserving targeted genome editing for candidates supported by strong functional evidence.^11^ Importantly, managing growth–stress trade-offs must be prioritized to ensure yield stability under stress conditions, with regulators such as *WRI1*, *SnRK2*, and *DELLA* proteins playing critical roles in balancing metabolic and developmental processes.^9^ Exploitation of natural genetic variation, particularly through interspecific hybrids (*E. guineensis × E. oleifera*), further expands the pool of adaptive traits.^92^ Combining this genetic diversity with standardized cold phenotyping and multi-omics profiling could facilitate the identification of favorable alleles and physiological traits for early selection.^15^ Advances in systems biology and AI-driven predictive modeling could further integrate genomic, molecular, and phenotypic information to predict favorable gene combinations and prioritize candidates for experimental validation.^117^ Ultimately, future progress will depend on integrating mechanistic insights with predictive and translational approaches, enabling the rational development of oil palm cultivars capable of maintaining productivity under sub-optimal temperatures. Rather than relying on a single technology, a staged strategy that combines feasible near-term breeding tools with progressively more advanced functional and genome-editing approaches is likely to provide the most realistic pathway toward cold-resilient oil palm.^11^ This integrative strategy supports the sustainable expansion of oil palm cultivation into cooler regions while maintaining yield and resilience under changing climatic conditions.

(A) Exploration of germplasm resources and natural genetic variation, including interspecific hybridization between *E. guineensis* and *E. oleifera*, as a foundation for identifying cold-tolerant traits. (B) Application of advanced molecular breeding tools such as CRISPR/Cas9-mediated gene editing (targeting key stress-responsive genes including *EgCBF, EgICE1, EgNCED, EgSAD*, and *EgFAD*) and marker-assisted/genomic selection to develop elite cultivars. (C) Integration of multi-omics technologies (transcriptomics, metabolomics, proteomics) with high-throughput phenotyping techniques (thermal imaging, chlorophyll fluorescence, and hyperspectral analysis) to guide precision breeding decisions. (D) Implementation of systems biology and AI-driven predictive modeling combining multi-omics data, transcription factor networks, and physiological responses to predict optimized cultivars with improved cold tolerance, enhanced growth stability, and reduced stress-induced trade-offs.

## 5. Conclusions

This review establishes that cold sensitivity in oil palm (*E. guineensis*) arises primarily from regulatory inefficiency rather than the absence of conserved stress-responsive pathways. Although key cold-response networks are present, their delayed activation and weak coordination constrain effective acclimation, leading to inadequate membrane stabilization, disrupted water relations, and insufficient antioxidant protection. These findings indicate that cold tolerance in oil palm is governed by the integration of signaling, transcriptional regulation, and metabolic adjustment, rather than by individual genes or isolated pathways. A critical implication is that improving cold resilience will require targeting regulatory hubs and network interactions instead of single-gene modifications. Comparative insights from temperate oilseed crops further demonstrate that effective cold adaptation depends on rapid signal propagation, sustained transcriptional activity, and coordinated physiological responses across osmotic, redox, and hormonal systems. However, a major challenge remains in translating mechanistic understanding into practical breeding outcomes. Addressing this gap will require the integration of multi-omics datasets with high-resolution phenotyping and predictive computational modeling to identify robust genetic targets and optimal gene combinations. In this context, genome editing and genomic selection offer significant potential, although their success will depend on the development of efficient functional validation systems in oil palm. Overall, advancing cold tolerance will require a transition toward systems-driven, design-oriented strategies that integrate genetics, physiology, and data science to enable the development of climate-resilient cultivars capable of sustaining productivity under low-temperature stress.
